# Detecting bipolarity using the Lebanese Arabic hypomania checklist (HCL-32): validation of shortened HCL versions

**DOI:** 10.3389/fpsyt.2026.1817223

**Published:** 2026-05-20

**Authors:** Josleen Al Barathie, Melissa Barakat, Elie Hantouche, George E. Karam, Elie G. Karam

**Affiliations:** 1 Institute for Development, Research, Advocacy, and Applied Care (IDRAAC), Beirut, Lebanon; 2 Department of Psychiatry and Clinical Psychology, Saint George University of Beirut Faculty of Medicine, Beirut, Lebanon; 3 Anxiety & Mood Center (CTAH), Paris, France; 4 Department of Psychiatry and Clinical Psychology, Saint George Hospital University Medical Center, Beirut, Lebanon

**Keywords:** bipolar disorder, hypomania checklist, major depressive disorder, short form, validation

## Abstract

**Introduction:**

Due to the under diagnosis of bipolar disorder, screening instruments such as the hypomania checklist 32 items (HCL-32) is used to differentiate between Bipolar Disorder (BD) and Major Depressive Disorder (MDD). However due to its lengthy format, efforts were done to validate a shorter alternative without compromising its ability to differentiate between BD and MDD. We aimed to shorten the HCL-32 and assess the screening performance of the three Lebanese Arabic abbreviated HCL versions (HCL-20, -16, and -8) relative to the full HCL-32 in a sample of clinically diagnosed patients with BD and MDD in Lebanon.

**Methods:**

In a sample of 760 patients (BD-I=29, BD-II=142, MDD=589) clinically diagnosed with BD and MDD, the screening performance of the three Lebanese Arabic abbreviated HCL versions (HCL-20, -16, and -8) as well as the full HCL-32, was assessed, looking at the reliability, sensitivity, and specificity.

**Results:**

All the shortened HCL versions showed strong reliability (a=0.78-0.90.) They also demonstrated good screening ability (AUC=0.8520- 0.8835) in differentiating BD from MDD. For the sensitivities across the shortened versions, they were consistently higher in BD-II vs MDD compared to BD-I vs MDD across all scales showing that the shortened versions have the ability to detect BD-II cases much more effectively.

**Discussion:**

This study is the first to validate the shortened HCL versions in an Arabic speaking population. The HCL- 16 appears to be the most optimal shortened scale for distinguishing between BD versus MDD. However, these findings should be interpreted in light of the study’s limitations including the use of retrospective data collection and item interdependence of the HCL-32.

## Introduction

Bipolar disorders (BD), as per the DSM-5, are mental health conditions involving mood, energy, and activity fluctuations ranging from very “up,” elevated, irritable, or energized state known as manic/hypomanic (highs) episodes to very “down,” sad, indifferent, or hopeless moods known as depressive (lows) episodes (1). BD has many subtypes. Bipolar I subtype is characterized by at least one full manic episode with or without depression while bipolar II subtype is characterized by hypomania with depression (2).

Clinicians continue to face difficulties in diagnosing BD early on. Evidence indicates that nearly 30% of individuals with BD wait close to a decade before receiving an accurate diagnosis (3–5) and initiating suitable treatment. BD are often overlooked in favor of major depressive disorder (MDD), with around 50–75% of patients with BD are misdiagnosed as MDD at first (57, 58) and BD-II being especially prone to under-recognition (6–8). This occurs because patients with BD-II commonly present during depressive episodes (9), while past (hypo)manic symptoms can remain unnoticed unless specifically explored (8, 10). In fact, in individuals with BD‐II, depressive episodes occur more frequently than hypomanic ones, at an estimated ratio of 39:1 (11). Furthermore, individuals may not spontaneously report periods which are subjectively dominated by good mood, high energy levels, increased productivity or decreased need for sleep as those states feel pleasant (12, 13). Although hypomanic episodes do produce observable changes in behavior, these shifts are not severe enough to cause functional impairment, as per the definition (14).

Confusing BD for unipolar MDD is clinically consequential. It can worsen prognosis, delay recovery due to ineffective treatment (9), worsen outcome due to higher rates of rehospitalization (9), increase recurrence, and contribute to antidepressant-induced mood elevation (9, 15). More seriously, studies have shown an increased risk of suicide in people with undiagnosed BD (3, 15).

To enhance BD identification, several screening instruments have been developed. The Mood Disorder Questionnaire (16)— a 13-item self-report measure assessing lifetime manic symptoms — is one of the most widely used ones. However, it has been shown to detect BD-II less effectively than BD-I (17, 18). The Bipolar Spectrum Diagnostic Scale (BSDS) (19) is another tool, which has its own limitations as well (20). This tool has a low specificity, where those with depression may be screened positive for bipolar disorder (20). Moreover, it has low discriminant ability in younger adults since the developed cut-offs were made for older adults (20). It also detects other emotional disorders which may mimic manic symptoms but are not reflective of bipolar disorder (20).

These limitations led Angst et al. to create the Hypomania Checklist-32 (HCL-32) (21), designed to better capture hypomanic features in individuals with MDD through specifically probing for behaviors, emotions and thoughts typically observed in hypomanic states (e.g. ‘I need less sleep’ or ‘I am less shy or inhibited’), and the questionnaire specifically refers to ‘a period when [you] were in a “high” state’. The questionnaire also includes items about the duration of such ‘highs’, as well as the current and habitual mood (21).

The usefulness of the HCL-32 has resulted in several published studies using clinical and non-clinical samples aiming at its translation and validation across many languages and countries including Italian, Chinese, German, Arabic, Taiwanese, Spanish, Farsi, Portuguese, Brazilian, and English speaking samples (UK) (18, 22–33). Notably, the original study identified a dual-factor model consisting of an “active/elated” dimension reflecting heightened energy and activity, and a “risk-taking/irritable” dimension reflecting impulsivity and engagement in hazardous behaviors (21). Afterwards, multiple studies replicated the original two-factor solution suggesting a sunny and dark side of hypomania (9, 18, 21, 26, 30, 32, 34–36), while some others have proposed three- or four-factor alternatives (28, 31, 33).

One of the studies that supported the two factor model is a major study on 5635 patients of whom 16.0% were diagnosed with bipolar disorder and 84.0% with MDD from 18 countries in five cultural regions: Iberia, Central Europe, Eastern Europe, North Africa/Near East and the Far East (37).

Despite its advantages and large-scale replication in multiple languages, the length of the HCL-32 has been viewed as a drawback, potentially limiting broader use (38). The scale may be impractical in fast-paced clinical environments or large epidemiological studies where shorter instruments reduce respondent burden and improve completion rates. Indeed, concerns about length contributed to the scale not being recommended in certain evidence-based guidelines, such as those by the National Institute of Health and Care Excellence (39).

Consequently, several efforts have focused on developing more concise versions of the HCL-32. A recent systematic review has identified 18 shortened versions, ranging from 8 to 31 items (40): Out of those short versions, two, both the validated and reasonable brief HCL‐16 and HCL‐20, seem to be most appropriate for clinical practice and research. Although the HCL‐28 performs well, but it was only slightly shortened compared with the original HCL‐32.

Forty et al. created the 16-item version using inter-item correlations to remove redundant content and found that those 16 items maintained similar sensitivity, specificity, and the original two-factor structure in a small number of patients with BD (n=59, including BD-I, -II, and -NOS) and patients with MDD (n=76) in the UK (38). Bech et al. used item response theory and retained 20 items [the Mokken model (41)], that performed well compared to HCL-32 in a Danish sample consisted of 59 patients with BD-I and 63 patients with unipolar depression (42). These efforts to shorten the HCL-32 created the foundation for subsequent cross-validation studies assessing how well those two shortened versions perform in multiple samples and identifying which one has the most optimal screening abilities. One effort by Meyer, Castelao (43) aimed at comparing the HCL-16, HCL-20, and HCL-32 in a Swiss epidemiological cohort and found the screening abilities of all the three scales comparable. Kim et al. also evaluated the HCL-16 and HCL-20, and further incorporated the HCL-8 due to its brevity (44). The HCL-8, shortened by Mosolov, Ushkalova (29) using multivariate logistic regression, demonstrated improved sensitivity without substantially reducing specificity in a sample of 147 patient with BD-II and 242 Russian patients with MDD. In this cross-cultural validation, the HCL-20 seems to be the provisionally optimal shortened version, demonstrating the most robust psychometric properties.

In contrast to the extensive validation literature of the full HCL-32 and the preliminary cross−cultural validations of the abbreviated HCL versions in international samples, evidence for Arabic dialects is notably scarce.

One study used Standard Arabic to validate the second revision of the HCL−32 (HCL−32−R2) among patients in Egypt and Saudi Arabia (23). ROC curve analysis revealed that the HCL-32-R2 was able to differentiate between patients with MDD and subthreshold BD with a good predictive validity according to the Area Under the Curve (AUC). This study supported a two-factor solution and reported good internal consistency of 0.85. However, there was a limited differentiation between (BD−II and MDD) and (BPI and MDD).

Although the latter study validated a standard Arabic version of the complete HCL, it is important to acknowledge that each Arabic-speaking nation has its unique dialect, and many individuals grasp concepts more precisely in their national dialect. Considering that the HCL 32 includes culturally sensitive items, it is imperative to validate the scale in national dialects to ensure accurate assessment and understanding of the items. For the long HCL version, only the validation of the Tunis Arabic dialect has been published (22). In this study, the HCL-32 was administered to 64 patients with MDD, 32 with BD, and 225 control subjects. The authors reported good reliability and stated that the bifactor implementation of the two-factor model demonstrated a good fit to the data. The HCL-32 succeeded in distinguishing patients with bipolar disorder BD from healthy controls (AUC: 73.4), as well as patients with BD from patients with MDD (AUC: 83.3). The sensitivity and specificity were 87% and 69% respectively.

Despite these validations of the full HCL 32 in Arabic, there is still a lack of literature on the validation of the full HCL-32 across different Arabic dialects and no literature at all on the abbreviated HCL versions (e.g., HCL 20, HCL 16, HCL 8) in any Arabic-speaking population. This highlights a substantial gap, given the considerable heterogeneity in dialects and cultural expressions of mood symptoms. We commenced this endeavor in this present study, by thoroughly assessing the screening performance of the three Lebanese Arabic abbreviated HCL versions (HCL-20, HCL-16, and HCL-8) relative to the full HCL-32 in a sample of clinically diagnosed patients with BD and MDD in Lebanon. Consistent with the original purpose of the HCL-32 and following the analytical approach of Kim et al., particular attention was given to the differentiation between BD-II and BD-I, given the former’s high risk of underdiagnosis.

## Methods

Data was obtained retrospectively from pre-collected medical records of new patients presenting for a psychiatric consultation in the outpatient psychiatry and psychology clinics at Saint George Hospital University Medical Center (SGHUMC) between 2015 and 2023. At SGHUMC’s psychiatry and psychology clinics, it is standard practice for patients to complete several screening instruments, including the HCL-32 (21). The Arabic HCL-32 was translated and back translated by two professional bilingual translators in 2015 using standard forward–backward translation procedures. Although this Lebanese version was used in routine clinical practice, it had not undergone a prior formal validation in a Lebanese population.

Following the screening instruments, a clinical diagnosis of BDI, BDII or MDD which is considered the gold standard is established through an in-person interview based on DSM criteria, first conducted by an experienced psychiatric nurse and then reviewed and confirmed by a psychiatrist.

For the current study, the included individuals are those who are adults ≥18 years, have completed the HCL-32 in Arabic, and diagnosed with MDD or BD. As for the exclusion criteria, it was those with incomplete records or those who filled the HCL-32 in English. This resulted in a sample size of seven hundred and sixty.

For descriptive statistics, mean and SD were reported for continuous data while N and % were reported for categorical data. For bivariate analysis, an ANOVA was conducted with a *post hoc* analysis where a significance level of 0.05 was used.

Reliability was assessed using Cronbach alpha where a Cronbach’s value of.90 and above is defined as excellent,.80-.89 as good, and.70-.79 as adequate (45).

For the criterion validity, The Receiver Operating Characteristic (ROC) curve was used to compare different HCL versions (full HCL-32 as well as its abbreviated versions). For items-specific inquiry, please refer to the **Supplementary Material** (**Supplementary Table 1**). The ROC was interpreted by the use of the AUC. According to Tape et al, AUC values of.70-.80 are defined as fair,.80-.90 as good, and.90- as excellent (46).

We also calculated sensitivity and specificity. Sensitivity is defined as “the proportion of true positives tests out of all patients with a condition” and specificity as “the percentage of true negatives out of all subjects who do not have a disease or condition” (47).

Lastly, in order to calculate the optimal cutoff for each HCL version, the Youden’s index was used, which is the cutoff point that maximizes the total of the sensitivity and specificity (48).

For the data analysis, Stata version 17 was used.

This study was approved by the Institutional Review Board (IRB) of the Faculty of Medicine at Saint George University of Beirut (SGUB), Beirut, Lebanon, which is registered with the U.S Office of Human Research Protections (OHRP) in the Department of health and Human Services.

## Results

### Demographic and clinical characteristics of the sample

Our study included a total of 760 patients, including 29 patients with BD-I, 142 with BD-II, and 589 with MDD (**Table 1**). The three diagnostic groups differed significantly in age, with both patients with BD-I and BD-II being younger than the MDD group (p <.001). Gender distribution varied across groups as well.

**Table 1:** Characteristics of the study sample (N = 760).

Variables	BDI (N=29)	BDII (N=142)	MDD (N=589)	P-value	*Post hoc* comparison
Age	33.07 (11.18)	32.32 (10.18)	40.5 (14.45)	<0.001	1 = 2<3
Gender (N,%)					
Female	13 (44.83%)	80 (56.34%)	308 (52.29%)	<0.001	
HCL32	18.41 (7.41)	17.83 (6.25)	6.09 (6.66)	<0.001	1 = 2>3
HCL20	11.44 (4.88)	11.25 (3.97)	3.86 (4.17)	<0.001	1 = 2>3
HCL16	8.13 (3.41)	8 (3.31)	2.5 (3.06)	<0.001	1 = 2>3
HCL8	5.03 (2.44)	4.61 (1.69)	1.72 (1.72)	<0.001	1 = 2>3

Across all versions of the HCL, *post-hoc* comparisons indicated that patients with BD-I and BD-II did not differ from each other on any HCL score, while both groups scored significantly higher than patients with MDD (p <.001) (**Table 1**).

### Reliability of the original and shortened HCL versions

Internal consistencies of the original and shortened scales are summarized in **Table 2**. The full HCL-32 demonstrated excellent reliability (Cronbach’s α = 0.94). The shortened versions also showed strong internal consistency, with a Cronbach’s α of 0.90 for HCL-20 and 0.87 for HCL-16. Reliability was lowest for the HCL-8 (α = 0.78), though still within an acceptable range.

**Table 2:** Cronbach alpha of the original and shortened scales.

Number of items	Reliability
HCL32	0.9375
HCL20	0.9012
HCL16	0.8653
HCL8	0.7754

### Screening performance of the shortened HCL versions compared with the original HCL-32

**Figure 1:**
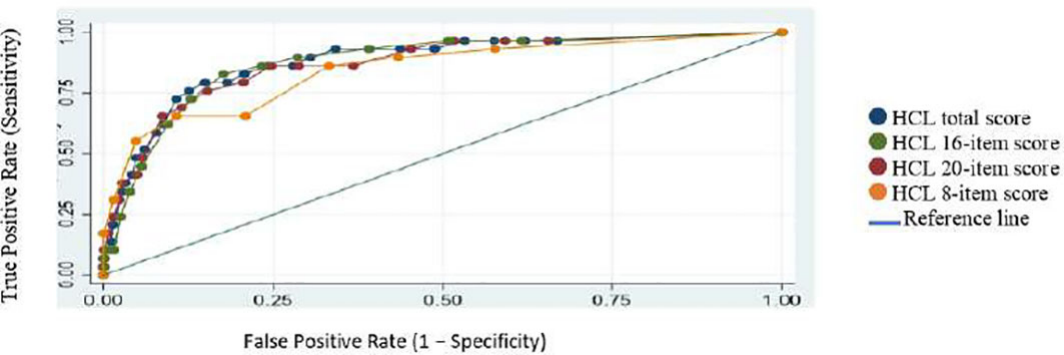
Receiver operating characteristic (ROC) curves comparing different HCL models (BDI combined vs MDD).

As shown in **Table 3**, all versions of the HCL demonstrated good screening ability in differentiating patients with bipolarity (BD-I and BD-II combined) from MDD. The AUC of the HCL-32 (AUC=0.8847) was high, and the AUCs of the shortened versions were very similar (HCL-20 = 0.8835; HCL-16 = 0.8751; HCL-8 = 0.8520). This was also evident in the ROC curves with large overlap across versions (**Figure 1**).

**Table 3:** Comparison of screening properties of the original and the shortened scales for discriminating between patients with any BP (N=171) and MDD (N=589).

Number of items	AUC [SE] (95%CI)	Threshold	Sensitivity	Specificity
HCL32	0.8847 [SE:0.01] (0.85-0.91)	≥14 ^a,b^	0.807	0.8501
HCL20	0.8835 [SE:0.01] (0.85-0.91)	≥8 ^a^	0.8421	0.7942
		≥10 ^b^	0.6842	0.8844
HCL16	0.8751 [SE:0.01] (0.84-0.9)	≥4 ^a^	0.924	0.7143
		≥8 ^b^	0.5439	0.9048
HCL8	0.8520 [SE: 0.01] (0.82-0.88)	≥3 ^a^	0.9064	0.6689
		≥5 ^b^	0.5789	0.893

^a^ optimal cutoff in our study.

^b^ optimal cutoff published.

Optimal cut-off scores for each scale were estimated in our sample, and previously published cut-off were also examined (**Table 3**). For the HCL-32, the optimal cutoff in our sample (≥14) was identical to the published value. Using this cut-off, the sensitivity was 80.7% while the specificity was 85.01%. For the shorter scales, the optimal cutoffs in our study were consistently lower than the published ones. Accordingly, diagnostic performance differed depending on whether study-derived or published cut-offs were applied.

When applying the study-derived optimal cut-offs, all shortened versions demonstrated higher sensitivity compared to applying published cut-offs (for HCL-20: 84.21% vs 68.42%/for HCL-16: 92.4% vs 54.39%/for HCL-8: 90.64% vs 57.89%).

In contrast, when applying study-derived optimal cut-offs, all shortened versions demonstrated lower specificity compared to applying published cut-offs (for HCL-20: 79.42% vs 88.44%/for HCL-16: 71.43% vs 90.48%/for HCL-8: 66.89% vs 89.3%).

This pattern indicates that published cut-offs tend to favor specificity at the expense of sensitivity in this sample, whereas locally derived cut-offs improve sensitivity but reduce specificity.

### Differential screening ability across bipolar subtypes

**Figure 2:**
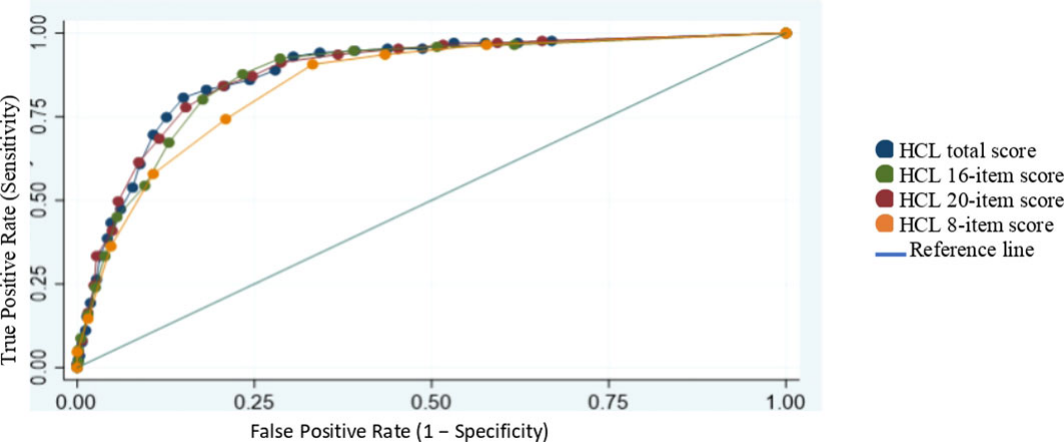
Receiver operating characteristic (ROC) curves comparing different HCL models (BD combined vs MDD).

For BD-I, AUCs for the different versions are 0.8798, 0.8701, 0.8771, and 0.8422 for HCL-32, -20, -16 and -8, respectively. The crude numbers show a huge overlap between the versions except for HCL-8 which has the lowest AUC. This was also evident in the ROC curves in **Figure 2** where HCL-8 has a clear deviation.

**Figure 3:**
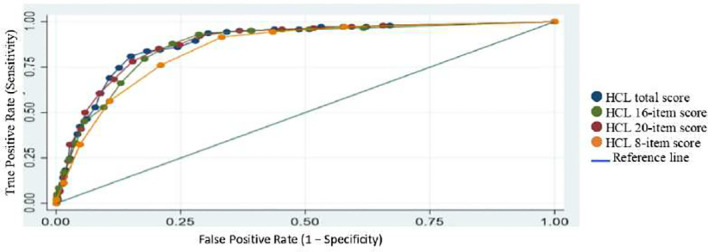
Receiver operating characteristic (ROC) curves comparing different HCL models (BDII combined vs MDD).

As for BD-II, AUCs ranged from 0.8536–0.8860 with similar trends to that seen in BDI (**Table 4**; **Figure 3**).

**Table 4:** Differential screening ability of the original and shortened versions of HCL in distinguishing between BPI (n=29) or BPII (n=142) against MDD (n=589).

Measure	HCL32	HCL20	HCL16	HCL8
AUC [SE] (95%CI)				
BD vs MDD	0.8847 [SE:0.01] (0.85-0.91)	0.8835 [SE:0.01] (0.85-0.91)	0.8751 [SE:0.01] (0.84-0.9)	0.8520 [SE: 0.01] (0.82-0.88)
BDI vs MDD	0.8798 [SE:0.03] (0.81-0.94)	0.8701 [SE:0.03] (0.8-0.93)	0.8771 [SE:0.03] (0.81-0.94)	0.8422 [SE: 0.04] (0.76-0.92)
BDII vs MDD	0.8536 [SE: 0.01] (0.85-0.92)	0.8860 [SE: 0.01] (0.85-0.91)	0.8745 [SE: 0.01] (0.84-0.9)	0.8536 [SE: 0.01] (0.82-0.88)
Optimal Cutoff	≥14 ^a,b^	≥8 ^a^	≥4 ^a^	≥3 ^a^
Sensitivity				
BD vs MDD	0.807	0.8421	0.924	0.9064
BDI vs MDD	0.7931	0.7931	0.8966	0.8621
BDII vs MDD	0.8099	0.8521	0.9296	0.9155
Specificity				
BD vs MDD	0.8501	0.7942	0.7143	0.6689
BDI vs MDD	0.8501	0.7942	0.7143	0.6689
BDII vs MDD	0.8501	0.7942	0.7143	0.6689

^a^ optimal cutoff in our study.

^b^ optimal cutoff published.

Looking at BD subtypes and using our cutoffs, sensitivities were consistently higher in BD-II vs MDD compared to BD-I vs MDD across all the shortened versions, whereas specificities remained stable (**Table 4**). These findings suggest that shortened versions have the ability to detect BD-II cases much more effectively than the BDI cases and even better than the original HCL-32.

## Discussion

In this study, we evaluated the psychometric properties and screening performance of the full HCL-32 and three abbreviated versions (HCL-20, HCL-16, and HCL-8) in a clinical sample of Lebanese patients with bipolar disorder (BD) and major depressive disorder (MDD). Compared to the original scale, the shortened versions retained good reliability and validity when differentiating BD (both type I and type II) from MDD. In all the versions, sensitivities were consistently higher for BD-II versus MDD compared to BD-I versus MDD, which emphasized the excellent use of the shortened HCL scales in detecting patients with BPII.

Cronbach alpha in our sample (HCL-32: α=0.9375; HCL-20: α=0.9012; HCL-16: α=0.8653; HCL-8: α=0.7754) were higher than those reported in prior studies. The original article of HCL-32 reported an internal consistency of 0.82 using Swedish and Italian samples (Angst, 2005), while subsequent studies on HCL-32 reported a Cronbach alpha between 0.86 to 0.94 (18, 34). For HCL-16, the reported alpha was 0.77 in a sample of individuals from UK (38) while Mosolov, Ushkalova (29) reported an alpha of 0.71 for the HCL-8 in a Russian sample.

The optimal cutoff point for the HCL-32 has most commonly been set at 14, as proposed in the original paper and supported by many subsequent validation studies (18, 28, 32, 33, 49–53).

However, alternative cutoff points have also been reported, including 12 (27, 35), 15 (54), 18 (34), 20 (55), and 8 (56). In our clinical sample, the sample’s cutoff was in alignment with the HCL-32 original cutoff of 14, and it yielded a sensitivity of 80.7%, closely aligning with the original report (80% for BP vs MDD). Looking at the specificity, it was much higher in our study reaching 85.01% versus 51% in the original study.

Consistent with Kim et al. (44), our findings raise concerns regarding the clinical utility of the HCL-8. In the study done by Kim et al, the HCL-8 was excluded due to its suboptimal reliability (α=0.60) and insufficient sensitivity. Even though the reliability of HCL-8 in our study was higher than that in Kim’s (α=0.7754), it remained inferior to both HCL-20 and HCL-16.

Following the lead of Kim et al, we performed a separate evaluation of BD-I versus MDD and BD-II versus MDD across all HCL versions (44). Sensitivity was consistently highest for BD-II versus MDD, particularly for the shortened versions (HCL-32: 80.99%; HCL-20: 85.21%; HCL-16: 92.96%; HCL-8: 91.55%). These findings reinforce the strength of the HCL, especially its abbreviated forms, in identifying BD-II cases, which are frequently underdiagnosed due to subtler hypomanic symptoms. This is an advantage of our study as prior studies have either examined all BD types together or focused specifically on BD-I, which does not resemble real clinical scenarios.

In order to have an optimal shortened version and in alignment with the goals of screening instruments contexts, sensitivity should be prioritized as it reduces false negatives avoiding missed BP diagnoses. Although the AUC values across the HCL-32 and its shortened versions were highly similar, indicating comparable overall discriminatory ability, AUC alone does not determine clinical utility. Instead, clinical applicability depends on the sensitivity–specificity balance at specific cut-off points. In this context, the HCL-16 outperformed both the HCL-20 and HCL-8 in identifying true cases, demonstrating the highest sensitivity across all comparisons, including all BD versus MDD (92.4%), BDI versus MDD (89.66%), and BD-II versus MDD (92.96%). Even if there is a high risk associated with false negatives, the slightly lower specificity of the HCL-16 (71.43%) as compared to the HCL-20 (79.42%) is clinically acceptable. Moreover, the HCL-16 maintained good internal consistency.

Therefore, the maximized sensitivity and acceptable specificity and reliability of the HCL-16, as shown in the above-mentioned findings, suggest that the HCL-16 offers an optimal balance for Lebanese clinical settings. In this way, the underdiagnosis of BD, especially BD-II, can be reduced.

Several limitations should be acknowledged. This study used retrospectively collected data from medical records of an outpatient psychiatry and psychology clinical sample. Another limitation is related to item interdependence of HCL-32 which might have influenced the results of the shortened versions. Thus, the shortened versions should be administered separately in future studies to confirm the findings of the present study. Lastly, exclusion of incomplete HCL-32 questionnaires, required for valid psychometric scoring, may limit the generalizability of results if missingness is not random.

## Conclusion

In summary, the Lebanese Arabic version of the HCL-32 and its shortened forms are reliable and valid screening tools for differentiating BD from MDD. This study is the first to validate the shortened HCL versions in an Arabic speaking population while distinguishing between BD-I and BD-II versus MDD. Among the shortened scales, the HCL-16 offers a practical and psychometric strong alternative to the full HCL-32 making it the most optimal scale to be used in clinical screening.

## Data Availability

The datasets presented in this article are not readily available because of privacy restrictions. Requests to access the datasets should be directed to egkaram@idraac.org.

## Glossary

ANOVA: Analysis of Variance
AUC: Area Under the Curve
BD: Bipolar Disorder
BD-I/BDI/BPI: Bipolar I Disorder
BD-II/BDII/BPII: Bipolar II Disorder
BP: Bipolar (bipolar disorder)
BSDS: Bipolar Spectrum Diagnostic Scale
CI: Confidence Interval
DSM: Diagnostic and Statistical Manual of Mental Disorders
DSM-5: Diagnostic and Statistical Manual of Mental Disorders, 5th edition
HCL: Hypomania Checklist
HCL-32: Hypomania Checklist – 32 items
HCL-20: Hypomania Checklist – 20 items
HCL-16: Hypomania Checklist – 16 items
HCL-8: Hypomania Checklist – 8 items
HCL-32-R2: Hypomania Checklist-32, second revision
IDRAAC: Institute for Development, Research, Advocacy, and Applied Care
IRB: Institutional Review Board
MDD: Major Depressive Disorder
OHRP: (U.S.) Office of Human Research Protections
ROC: Receiver Operating Characteristic
SD: Standard Deviation
SE: Standard Error
SGHUMC: Saint George Hospital University Medical Center
SGUB: Saint George University of Beirut
UK: United Kingdom
