# Supplementary material for: Detecting bipolarity using the Lebanese Arabic hypomania checklist (HCL-32): validation of shortened HCL versions
Source: Front Psychiatry. 2026 May 20;17:1817223. doi: 10.3389/fpsyt.2026.1817223 (PMC13229889; doi:10.3389/fpsyt.2026.1817223)
Supplement: Supplementary file 1 [file Table1.docx]

**Supplementary Table S1:** Items of the original Hypomania Checklist-32 (HCL-32) and their inclusion across the three validated short versions: HCL-20 (Bech et al., 2011), HCL-16 (Forty et al., 2010), and HCL-8 (Mosolov et al., 2014). The table indicates which HCL-32 items are retained in each abbreviated version.

**Table S1.** Items belonging to the three short versions of the Hypomania Checklist-32 (HCL- 32)

| HCL-32 items | HCL-20  (Bech et al., 2011) | HCL-16  (Forty et al., 2010) | HCL-8  (Mosolov et al., 2014) |
| --- | --- | --- | --- |
| 1. I need less sleep | ✘ | ✘ | ✘ |
| 2. I feel more energetic and more active | ✘ |  |  |
| 3. I am more self-confident |  |  |  |
| 4. I enjoy my work more | ✘ | ✘ |  |
| 5. I am more social (make more phone calls, go out more) | ✘ |  |  |
| 6. I want to travel and/or do travel more |  | ✘ |  |
| 7. I tend to drive faster or take more risks when driving | ✘ |  |  |
| 8. I spend more money/too much money | ✘ | ✘ |  |
| 9. I take more risks in my daily life (in my work and/or other activities) | ✘ | ✘ |  |
| 10. I am physically more active (sports etc.) | ✘ | ✘ |  |
| 11. I plan more activities or projects | ✘ |  |  |
| 12. I have more ideas, I am more creative |  |  |  |
| 13. I am less shy or inhibited |  | ✘ | ✘ |
| 14. I wear more colorful and more extravagant clothes/make up |  | ✘ |  |
| 15. I want to meet or actually do meet more people | ✘ |  |  |
| 16. I am more interested in sex, and/or have an increased sexual desire |  |  |  |
| 17. I am more flirtatious and/or am more sexually active |  | ✘ |  |
| 18. I talk more | ✘ |  | ✘ |
| 19. I think faster |  | ✘ |  |
| 20. I make more jokes or puns when I am talking | ✘ | ✘ | ✘ |
| 21. I am more easily distracted | ✘ |  | ✘ |
| 22. I engage in lot of new things |  |  |  |
| 23. My thoughts vary from topic to topic | ✘ |  | ✘ |
| 24. I do things more quickly and/or more easily |  |  |  |
| 25. I am more impatient and/or get irritable more easily | ✘ |  |  |
| 26. I can be exhausting or irritating for others |  |  | ✘ |
| 27. I get into more quarrels | ✘ | ✘ |  |
| 28. My mood is higher, more optimistic | ✘ | ✘ | ✘ |
| 29. I drink more coffee | ✘ |  |  |
| 30. I smoke more cigarettes |  | ✘ |  |
| 31. I drink more alcohol | ✘ | ✘ |  |
| 32. I take more drugs (sedatives, anxiolytics, stimulants) | ✘ | ✘ |  |
